# Optical coherence tomography findings in patients prior to cataract surgery regarded as unremarkable with ophthalmoscopy

**DOI:** 10.1371/journal.pone.0208980

**Published:** 2018-12-11

**Authors:** Antonia Kowallick, Charlotte Viola Fischer, Hans Hoerauf

**Affiliations:** Department of Ophthalmology, University Medical Center Goettingen, Germany; Massachusetts Eye & Ear Infirmary, Harvard Medical School, UNITED STATES

## Abstract

**Purpose:**

To investigate the feasibility and diagnostic benefit of routinely performed preoperative macular spectral-domain (SD-) optical coherence tomography (OCT) for the detection of macular pathology in patients with normal biomicroscopic funduscopy prior to cataract surgery.

**Methods:**

Prospective, single center study. A total of 162 eyes of 123 consecutive patients referred for cataract surgery with a visual acuity better than 20/100, absence of macular symptoms such as metamorphopsia, no history of previous intravitreal procedures and a normal funduscopic appearance on biomicroscopy underwent an additional SD-OCT-examination prior to cataract surgery. OCT-scans were classified in three categories: normal, degenerative vitreous changes without impact on visual outcome or pathological with potential impact on visual outcome.

**Results:**

80 eyes (49.38%) showed normal OCT-scans. 69 eyes (42.59%) were classified as degenerative vitreous changes without impact on visual outcome and 20 eyes (12.35%) as pathological with potential impact on visual outcome. The indication of cataract surgery or the therapeutic strategy remained unchanged in all patients. In patients with pathological alterations further follow-up examinations were recommended.

**Conclusions:**

Routine SD-OCT-imaging of the macular region in patients prior to cataract surgery was feasible to detect macular pathologies in a considerable number of patients, which remained undiagnosed on biomicroscopic funduscopy. Although OCT-findings did not impact therapeutic strategy in this study, preoperative judgement of the expected visual outcome and patient´s informed consent can improve.

## Introduction

Cataract is one of the most common reasons for visual loss in elderly people. However, a considerable number of patients with cataract exhibit other co-existing ocular diseases including macular pathologies.[1] Macular examination routinely performed by biomicroscopic funduscopy is one of the standard elements of preoperative cataract assessment. Due to related media opacity, poor pupil dilatation or photophobia, subtle macular pathologies may be missed using ophthalmoscopic techniques[2–4], although co-existing macular pathologies might have important prognostic implications. Furthermore, co-existing pathologies might result in incorrect treatment decisions or unrealistically high expectations for postoperative visual outcome which may cause postoperative patient dissatisfaction or even legal consequences. Particularly in patients undergoing refractive surgery, the implantation of multifocal intraocular lenses may be contraindicated in case of co-existing macular pathology.[5] Spectral-Domain (SD-) Optical coherence tomography (OCT) is an imaging technique that allows for a detailed cross-sectional evaluation of the macular region even in the presence of media opacity or narrow pupil. It has the potential to improve preoperative diagnostics in patients prior to cataract surgery with considerable impact on further therapy.[2–5] Accordingly, the aim of the current study was to evaluate the feasibility and diagnostic benefit of routinely performed preoperative macular OCT-imaging in patients prior to cataract surgery presenting with normal biomicroscopic funduscopy and without visual symptoms typical for macular diseases.

## Methods

This single-center, prospective study was conducted at the Department of Ophthalmology at University Medical Centre of Goettingen, Germany. It adheres to the tenets of the Declaration of Helsinki and was approved by the local ethics committee (Research Ethics Committee, University of Goettingen). Consecutive patients being referred from resident non-surgical ophthalmologists for cataract surgery to the outpatient department of the University Eye Clinic Goettingen without documented retinal diseases were recruited. Informed consent was obtained from all individual participants included in the study. Patients with known coexisting retinal pathologies, current macular or retinal visual symptoms, a history of vitreoretinal procedures as well as patients with a best-corrected visual acuity worse than 20/100 were excluded (see Table 1). All participants underwent a comprehensive ophthalmological examination including ocular biometry, visual function, slit-lamp-biomicroscopy, a dilated examination of the central and peripheral retina with indirect and direct ophthalmoscopy by a resident as well as a consultant ophthalmologist. In total six residents and four consultant ophthalmologists participated in these study investigations to simulate best real-life conditions of examiners with different and variable diagnostic abilities. If fundus biomicroscopy of the referring ophthalmologist as well as of one resident and one consultant of our clinic revealed no pathological findings, an additional SD-OCT of the macular region with five horizontal and five vertical scans was performed using a Cirrus High-Definition SD-OCT (Carl Zeiss Meditec, Jena, Germany). SD-OCT measurements were always performed prior to cataract surgery and OCT readings were done by an independent consultant blinded to patients history and funduscopic findings. OCT-scans were classified as a) normal, b) with degenerative vitreous changes without impact on visual outcome or c) pathological with potential impact on visual outcome. In the majority of patients (n = 106) OCT was performed on the same day immediately after funduscopic examination, in 26 patients the mean time interval between funduscopic and OCT-examinations was 51 (3–111) days.

**Table 1:** Exclusion criteria.

macular symptoms (e.g. metamorphopsia, micropsia) clinical exclusion citeria: • macular edema • retinal vascular changes (e.g. micro-/macroaneurysms, haemorrhages, edema) • retinal exsudates • exsudative age-related macular degeneration changes (e.g. Drusen, retinal pigment epithelium changes or atrophy, choroidal neovascularization) • Vitreoretinal disorders (e.g. epiretinal gliosis, cellophane maculopathy, vitreomacular traction, macular hole)
history of vitreoretinal procedures (including intravitreal injections)
best-corrected visual acuity less than 20/100
age < 18 years

## Results

174 eyes of 133 patients who met the inclusion criteria were enrolled in the study. 12 eyes of 10 patients were excluded from the study due to poor OCT-image-quality resulting in a total number of 162 eyes (77 right and 85 left) of 123 patients. The mean age was 71.9 years (range 38–91 years), 53.7% were male. Visual acuity ranged from 20/20 to 20/100 with a median of 20/40. 21 patients (11 male) suffered from diabetes mellitus. Clinical examinations and OCT-imaging were performed on the same day except for 26 patients. In these 26 patients, there was a delay of 51 days on average between OCT-imaging and clinical examinations (range 3–114 days).

80 eyes (49.38%) showed no pathology on OCT-images. 69 eyes (42.59%) were classified as degenerative vitreous changes without impact on visual outcome and 20 eyes (12.35%) as pathological with potential impact on visual outcome. Cystoid macular edema, macular hole, or exudative age-related macular degeneration were detected in none of the cases. OCT-findings are summarized in Table 2.

**Table 2:** Summary of OCT-findings.

OCT-finding	Number of eyes (n = 162)	Ø age (years)
Unremarkable	80 (49.38%)	71.7
OCT-findings without impact on visual outcome	69 (42,59%)	73
changes at the vitreoretinal interface		
detachment in the perimacular region	11 (6.79%)	67.7
vitreomacular adhesion	6 (3.70%)	68.8
vitreofoveal adhesion	22 (13.58%)	72.7
vitreopapillary adhesion	27 (16.66%)	75.6
vitreous opacity	3 (1.85%)	75.7
Pathological with potential impact on visual outcome utome	20 (12.35%)	74.6
epiretinal membrane	10 (6.17%)	68.9
perimacular/foveal drusen	4 (2.47%)	83.8
fovea plana	3 (1.85%)	77.5
atrophy of retinal pigment epithelium	1 (0.62%)	
chorioretinal scar	1 (0.62%)	
pseudovitelliform lesion	1 (0.62%)	

### OCT-findings of the vitreoretinal interface without impact on visual outcome

**Fig 1:**
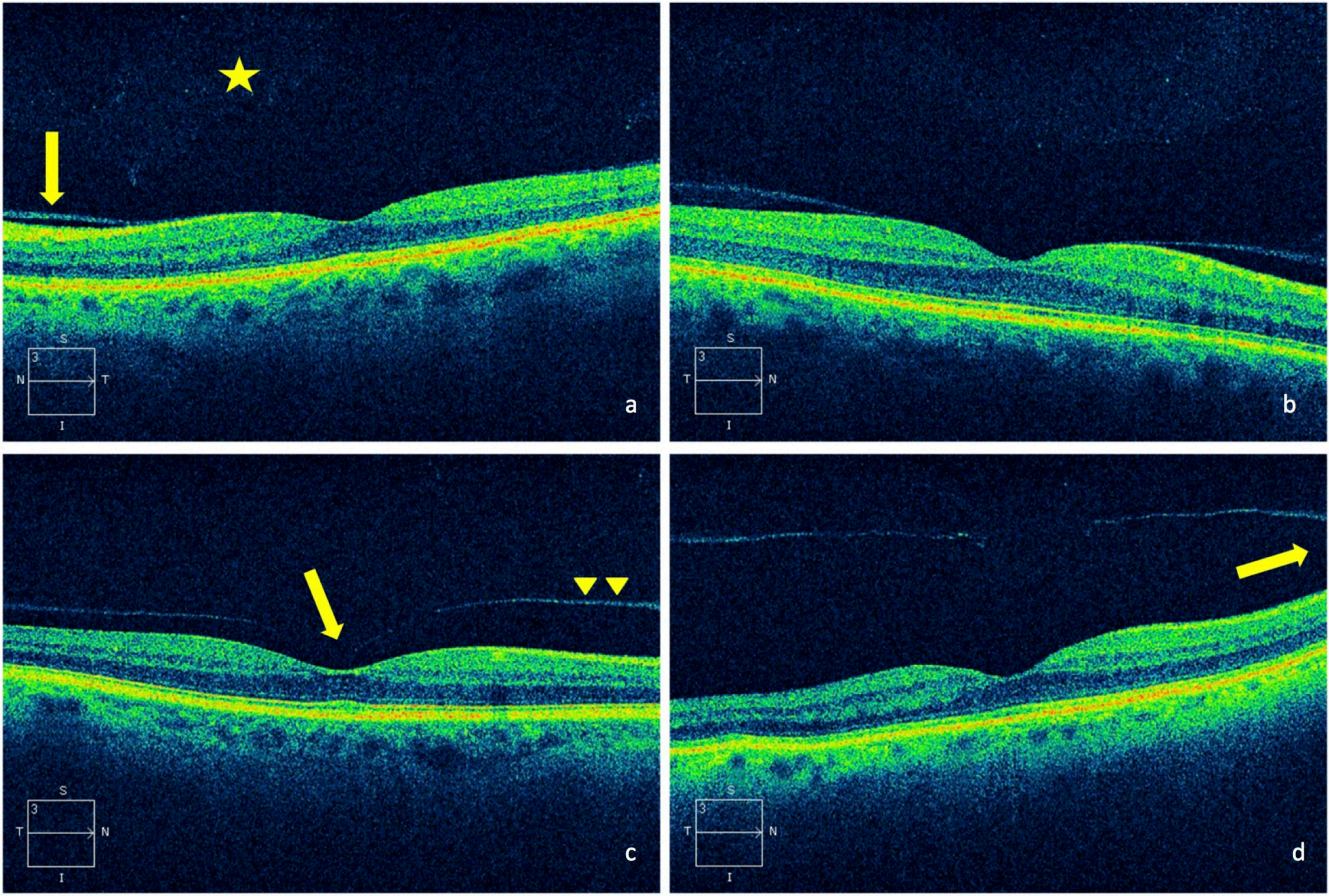
SD-OCT-images of different degenerative posterior vitreous changes. a. shallow posterior vitreous detachment in the perifovea (arrow), discrete vitreal opacities (*), b. vitreomacular adhesion, c. vitreofoveal adhesion (arrow) with posterior hyaloid near the retinal surface (arrowheads) still attached at the optic nerve head, d. vitreopapillary adhesion (arrow), the posterior hyaloid slightly detached from the fovea.

66 eyes showed posterior vitreous detachment at the vitreoretinal interface identified as a thin hyperreflective signal anterior the nerve fiber layer. With increasing age of patients, the prevalence of posterior vitreous detachment raised. 13 patients showed the same stage of posterior vitreous detachment in both eyes. In 11 eyes a localized detachment over the perimacular region (Fig 1A) was identified. 28 eyes showed a vitreomacular (6/28) or a vitreofoveal adhesion without traction (22/28) (Fig 1B and 1C). A vitreopapillary adhesion was observed in 27 eyes (Fig 1D).

### Pathological OCT-findings with potential influence on visual outcome

**Fig 2:**
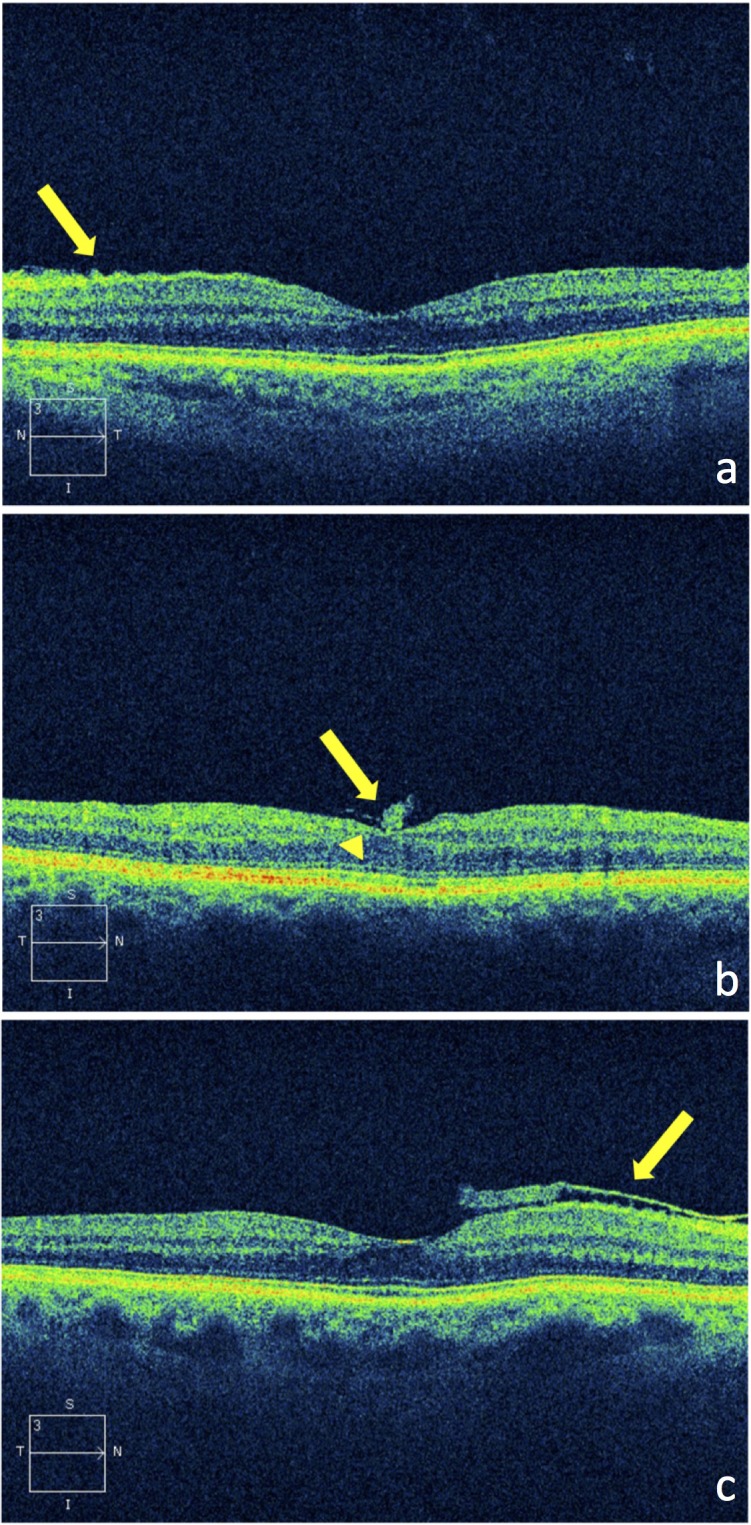
SD-OCT-images of epiretinal membranes. a. with retinal puckering (arrow), b. with foveal elevation (arrowhead) and gliotic tissue (arrow), c. lying on top of the retina (arrow), without attendant visible traction.

In 10 eyes of 9 patients an epiretinal membrane (ERM) without traction on the retina was diagnosed in OCT. 9/10 ERM were completely adherent to the retina. Of these nine ERM, five were identified due to retinal puckering (Fig 2A), two ERM caused foveal elevation (Fig 2B) and two ERM were detected due to higher reflectivity but revealed no retinal changes. Fig 2C shows the only 1/10 ERM in this study which appeared as a separate thin superficial hyperreflective layer without causing retinal changes.

**Fig 3:**
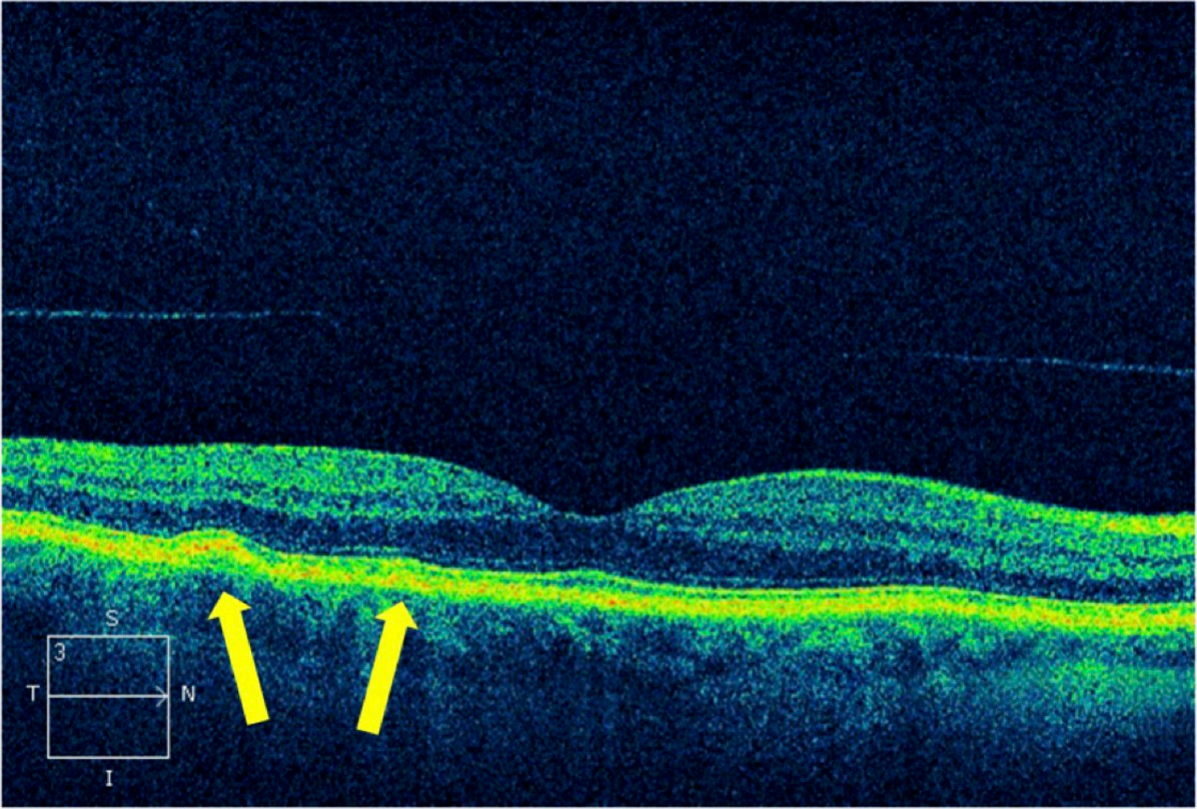
SD-OCT-image of perifoveal soft drusen (arrows) with normal overlying neurosensory retina.

Furthermore, SD-OCT-imaging revealed drusen in 4 eyes which were not seen by funduscopy. In 2/4 eyes, soft drusen were located subfoveally without other retinal changes (Fig 3).

Surprisingly, 3 eyes (2 patients) showed–even after thorough adjustment of the section plane–no foveal depression. The patients did not show any further pathology like aniridia or hypopigmentation of the fundus and therefore were classified as an isolated form of fovea plana. [6] In 3 eyes OCT revealed other: central atrophy of the retinal pigment epithelium (n = 1), chorioretinal scaring (n = 1) and a pseudovitelliform lesion (n = 1). Although a total of 20 eyes showed macular pathologies, none of the findings had an impact on the therapeutic strategy. All patients were scheduled for cataract surgery without further therapeutic consequences. However, in affected patients the informed consent regarding a possible impact on visual prognosis after cataract surgery was adapted and patients were advised to make use of regular follow-up examinations of the fundus.

## Discussion

The presented study aimed to examine the feasibility and diagnostic benefit of routinely performed preoperative macular OCT for the detection of macular pathology in patients with normal funduscopic appearance prior to cataract surgery. The results of our study show that a considerable number of cases with subtle macular pathologies as diagnosed by OCT were missed despite comprehensive preoperative biomicroscopic funduscopy.

### Degenerative vitreous changes

SD-OCT is a very sensitive diagnostic method for the detection of early changes at the vitreoretinal interface.[7] In the presented study, 66/162 eyes showed posterior vitreous detachment at the vitreoretinal interface. Our study coincides with other studies that vitreous detachment is increasing with proceeding age.[8] However, even SD-OCT-imaging-technique has limitations to discriminate reliably between complete vitreoretinal adherence and a fully detached vitreous. 28/162 eyes showed vitreomacular or vitreofoveal adhesion without exerting visible traction. This exact diagnosis is certainly a strength of SD-OCT technique, but vitreomacular adhesion is not known as a risk factor for postoperative cystoid edema and thus had no influence on surgical policy.

### Epiretinal membrane

In the presented study, 10/162 eyes showed an ERM. Previous data suggest an increased prevalence of ERM in patients between 70–79 years because of vitreous liquefaction, previous lens extraction or retinal diseases.[9,10] The detection of ERM by biomicroscopic fundoscopy decreases with age caused by reduced fundus evaluation in cataractous eyes. Jahn et al. showed an increased prevalence of ERM detected by biomicroscopic fundoscopy seven days after cataract extraction compared to the preoperative situation, suggesting that the ERM were already present preoperatively, but undetected via biomicroscopic fundoscopy.[11] Contreras et al. demonstrated that integration of OCT in the preoperative workflow is able to detect biomicroscopically overlooked ERM.[12] It is commonly known, that ERM is slowly progressive and leads to visual impairment only in certain cases.[13] Nevertheless, recent studies reported that patients with ERM prior to cataract surgery have a higher risk for the development of a postoperative epimacular membrane rip and a postoperative cystoid macular edema with subsequent visual impairment.[14,15] Hence it would be desirable if affected patients can be adequately informed preoperatively about the possible influence on postoperative visual outcome and appropriate follow-up examinations. In presence of a cataract, a significant and symptomatic ERM might change the therapeutic strategy to a combined procedure including cataract extraction and vitrectomy with membrane peeling. However, in our study patients with undetected preoperative ERM did not complain about disturbing typical symptoms such as metamorphopsia and therefore the therapeutic strategy was not changed. It has to be kept in mind that an ERM may be missed even by SD-OCT-imaging. The additional judgement of perimacular vessel distortion by biomicroscopy or on the infrared image of the OCT may help to identify a flat ERM. It is well known that an ERM is a marked risk factor for the development of a pseudophakic cystoid macular edema. On the one hand, this appears important for the patient in terms of informed consent and further for the surgeon to avoid potential liability claims.[15]

### Drusen

Previous studies showed no significant superiority of SD-OCT over biomicroscopy regarding the detection of drusen.[16] In contrast, the current study was able to detect drusen in 4/162 eyes via OCT-imaging which were not detected by funduscopy. However, the study cohorts may not be comparable, since the patients in our study had a significant cataract obscuring fundus visualization. The importance of early diagnosis and treatment of exsudative age-related macular degeneration (AMD) particularly at early disease stages which are often only detectable by SD-OCT while visual acuity is still preserved is generally known.[17] Patients with soft drusen have a higher risk for the development of exsudative AMD.[18] Despite no relationship between the progression of AMD and cataract surgery was proven, it is particularly important to inform affected patients that this risk exist independently from cataract surgery.[19] Furthermore, the diagnosis of drusen identifies patients at risk for the development of an exsudative AMD and improves preoperative patient information regarding necessary follow up examinations.

### Other findings

In 3/162 eyes (2 patients) we could not identify a foveal depression despite the OCT measurement was performed thoroughly using vertical and horizontal scan distance of 0.25 mm. Since the postoperative visual outcome may be limited in patients with fovea plana the informed consent prior to cataract surgery has to be adapted.[20]

### Limitations of the study

According to the study protocol a dilated biomicroscopic funduscopy was performed by a referring ophthalmologist as well as a resident and a consultant in our clinic. Only if all three did not detect a pathology on funduscopy, patients were enrolled in the study. This situation is usually not given during routine preoperative assessment prior to cataract surgery. Moreover, the knowledge that patients could participate in this study, may have encouraged the examiners to a more thorough clinical examination and may lead to a more detailed observation. Therefore, in a real life setting an even higher percentage of undetected macular pathologies by funduscopy should be expected, which may result in a higher percentage of additional detected macular pathologies by OCT. However, it is also possible that there were false positive findings during biomicroscopic funduscopy, which might have resulted in a selection bias. The majority of patients underwent OCT measurements and funduscopy on the same day, except for 26 cases. Although most of the detected pathologies represent chronic conditions, we cannot rule out that some pathologies developed or proceeded during the time delay between both examinations. Furthermore, the time interval of few weeks between examination of the referring ophthalmologist and examination in our outpatient clinic may have played a role. Regarding the state of the posterior hyaloid it is important to highlight that a possible posterior vitreous detachment identified during biomicroscopic funduscopy might not have been documented. Therefore, it cannot be assessed exactly in how many cases posterior vitreous detachments were missed since progressive detachments of the posterior hyaloid cannot be identified by SD-OCT-images only. Post-surgical follow-up examinations have not been performed, accordingly, we are not able to correlate the visual outcome with our SD-OCT-findings and therefore cannot judge whether there was a clinically relevant influence, which should be addressed in future follow-up studies. It is important to note that at the time of OCT-examination all patients had a visual acuity better than 20/100. Whether or not the reported findings hold promise in case of dense cataract with visual acuity worse than 20/100 remains to be investigated. We had to exclude 12 eyes due to poor image quality because of dense cataract. In this regard, swept-source OCT might evolve its potential particularly in the presence of media opacities as discussed previously.[21,22] Another pitfall of the study is the limited number of recruited patients and consequently the proportion of undiagnosed pathologies may not be representative. In conclusion, SD-OCT appears as a sensitive preoperative diagnostic tool prior to cataract surgery in patients with normal funduscopic appearance. The results of the study depict that in the presence of cataract, a considerable number of cases with subtle macular pathology remained undiagnosed even by experienced ophthalmologists. Nevertheless, our study also demonstrates that patients prior to cataract surgery and without visual symptoms typical of macular pathology do not tend to have advanced macular pathologies. But it has to be considered that visual symptoms especially in early stages of macular pathology may not be recognized by all patients since they may be masked by the symptoms of the co-existing cataract. Although the OCT-findings did not impact on therapeutic strategy in this study, they markedly improved patient´s informed consent, the estimation of visual expectancy and possible predictive risk factors such as ERM. This assessment is particularly essential for an optimal selection and guidance of patients prior to refractive lens surgery and with high demands on visual outcome. This study is not able to prove that a preoperative SD-OCT prior to cataract surgery improves functional outcome since OCT-findings in this study had no influence on the therapeutic strategy and no postoperative assessment was performed. Thus, a future study with a prospective randomized design comparing the functional outcomes of surgery with and without a pre-operative OCT would be senseful. However, this study showed that subtle macular pathologies can be overlooked despite a thorough fundus biomicroscopy and a preoperative SD-OCT is able to improve the detection rate.
